# Smart Grid IoT Framework for Predicting Energy Consumption Using Federated Learning Homomorphic Encryption

**DOI:** 10.3390/s25123700

**Published:** 2025-06-13

**Authors:** Filip Jerkovic, Nurul I. Sarkar, Jahan Ali

**Affiliations:** 1 Department of Computer and Information Sciences, School of Engineering, Computer and Mathematical Sciences, Auckland University of Technology, Auckland 1010, New Zealand; jnx2090@autuni.ac.nz (F.J.); jahan.a@ica.ac.nz (J.A.); 2 Department of IT and Electrical Engineering, International College of Auckland, Auckland 1010, New Zealand

**Keywords:** federated learning (FL), internet of things (IoT), smart grid (SG), edge computing, machine learning (ML), internet privacy and security

## Abstract

Homomorphic Encryption (HE) introduces new dimensions of security and privacy within federated learning (FL) and internet of things (IoT) frameworks that allow preservation of user privacy when handling data for FL occurring in Smart Grid (SG) technologies. In this paper, we propose a novel SG IoT framework to provide a solution for predicting energy consumption while preserving user privacy in a smart grid system. The proposed framework is based on the integration of FL, edge computing, and HE principles to provide a robust and secure framework to conduct machine learning workloads end-to-end. In the proposed framework, edge devices are connected to each other using P2P networking, and the data exchanged between peers is encrypted using Cheon–Kim–Kim–Song (CKKS) fully HE. The results obtained show that the system can predict energy consumption as well as preserve user privacy in SG scenarios. The findings provide an insight into the SG IoT framework that can help network researchers and engineers contribute further towards developing a next-generation SG IoT system.

## 1. Introduction

Federated learning (FL) is a novel approach to securing machine learning (ML) by distributing workloads across multiple machines in a specific domain, allowing clients to train ML models collaboratively without exposing raw data [1]. Within the realm of SG infrastructure, the concept of FL is still novel, where the application has not been fully realized yet, including concerns around efficiency gains and security measures [2]. Nair et al. [3] proposed a privacy-preserving FL framework for IoMT that leverages edge computing to process sensitive medical data securely. Their approach ensures data privacy while enabling efficient distributed learning, highlighting the critical role of edge intelligence in safeguarding user information.

SG represents a significant evolution in energy management systems, integrating advanced technologies such as IoT, ML, and edge computing to optimize energy distribution and consumption. At present, there is wide industry adoption of IoT devices, such as smart meters, which automate the meter reading process for electricity providers through internet communication [3,4,5]. These devices collect vast amounts of data on energy consumption, which is then used to extrapolate power consumption patterns and make predictions for individual homes and commercial buildings. These predictions are critical for ensuring that power grid requirements are met on a city-wide scale, enabling utilities to balance supply and demand, reduce energy waste, and improve overall grid reliability [5]. Moreover, the implementation of IoT applications offers the interconnection of devices that collect and exchange data over the internet, enabling real-time monitoring and control [3,6]. In the context of SG, IoT devices such as smart meters play a critical role in automating energy consumption monitoring and optimization.

As more manual processes continue to be digitized within this domain, the use of data to make informed decisions through data-driven decision-making has become increasingly prevalent. However, this shift towards digitization and data reliance also introduces significant challenges, particularly data security and user privacy. For example, the detailed energy consumption data collected by smart meters can reveal intimate details about an individual’s lifestyle, habits, and daily routines [7,8,9]. This raises serious privacy concerns, as unauthorized access to such data could lead to misuse or exploitation. Moreover, the aggregation and analysis of such data on centralized servers create vulnerabilities, as these servers become attractive targets for cyberattacks, such as Distributed Denial of Service (DDoS) attacks or data breaches [10]. To address these challenges, there is a growing need for robust privacy-preserving techniques that can safeguard user data while still enabling the benefits of data-driven energy management. One such technique is HE, a cryptographic method that allows computations to be performed on encrypted data without the need for decryption [4,5,11]. This unique property of HE ensures that sensitive information remains secure throughout the data processing pipeline, making it particularly well-suited for applications in FL. By integrating HE into FL frameworks, it is possible to perform secure model aggregation and training without exposing raw data, thereby preserving user privacy while still achieving accurate and reliable predictions [12]. To build on the privacy preservation feature that FL offers, we introduce HE, a novel encryption scheme that behaves similarly to other encryption schemes, incorporating a key generation function, an encryption function, a decryption function, and an evaluation function as one of the core components of the FL framework [13]. The novelty in HE is that it allows the data to be manipulated while in an encrypted state, which means that the data is never exposed in a decrypted state, thus preserving the privacy and integrity of security of the data during the model aggregation process [13]. We include HE to improve the aspect of privacy preservation that P2PFL offers by incorporating it into model aggregation for federated energy consumption prediction models. Therefore, this paper aims to develop a novel IoT framework utilizing a combination of FL, Edge Computing (EC), and HE principles with the goal of predicting energy consumption patterns in an SG system while preserving user privacy End-to-End (E2E).

### 1.1. Research Challenges

A common challenge when working with ML is model tuning. An incorrectly tuned model overfits or underfits data, that can produce inaccurate predictions as the model fails to determine the true relationship of the data. Another challenge lies in utilizing HE to encrypt large amounts of data. While Cheon–Kim–Kim–Song (CKKS) and an HE schemes improve time complexity at the cost of accuracy, they still use a lot of memory when encrypting workloads, which is inefficient. Furthermore, a third challenge is the need to establish a secure communication protocol between peers in a secure P2P context, with a method of establishing P2P connections in the first place.

### 1.2. Research Scope and Contribution

The main contribution of this paper is the development and verification of an SG IoT framework for predicting energy consumption using peer-to-peer federated learning (P2PFL) and HE to preserve user privacy. This research offers a detailed review and analysis of existing frameworks and approaches to privacy preservation using FL and HE. We also explore the application of the framework in this space by developing a software simulation to evaluate the practicality of our framework in a real-world scenario. For the literature review, we looked at recent work over the last five years using well-known databases such as Google Scholar, Scopus, Elsevier Science Direct, and IEEE Xplore library. Search terms used in this paper include federated learning framework, smart grid federated learning framework, federated learning IoT framework, and machine learning for federated learning.

The main contributions of this paper are summarized as follows:

- We develop an algorithm for homomorphic encryption (HE) to preserve user privacy in smart grid (SG) systems. To this end, we optimize (predict) energy consumption using the FL model reported in Section 3.
- We develop an SG IoT framework by integrating P2PFL architecture and HE principles. To this end, we formulated mathematical models by deriving efficient FHE encryption through Equations (1)–(6) reported in Section 4.
- In the context of IoT, we explore a practical application of the framework with IoT devices involving SG for predicting energy consumption and optimization. To this end, we develop a simulation model for system performance evaluation and validation. We also contribute code (written in Python) for system design and evaluation purposes. The source code is publicly available (https://github.com/FilUnderscore/SG-P2PFL-HE (accessed on 10 April 2025)) for further exploration in the emerging field of SG IoT energy consumption.

### 1.3. Structure of the Article

The rest of this paper is organized as follows. The related work on FL frameworks in an SG setting, as well as the unification of P2PFL and HE, are presented in Section 2. The SG IoT framework design is discussed in Section 3. The system simulation, as well as an HE optimization, is presented in Section 4. The research design and methodology are discussed in Section 5. The system evaluation and test results are presented in Section 6; the practical implications are also discussed in this section. Finally, the paper is concluded in Section 7. Table 1 lists the abbreviations used in this paper.

## 2. Related Work

Federated learning, as a distributed machine learning paradigm, enables multiple parties to collaboratively train a model while maintaining the privacy of their raw data [1]. This approach has been examined across a range of application domains and has been outlined in terms of its types, simulation environments, and implementation challenges.

In smart energy systems, the adaptive federated learning methods have been developed for energy consumption forecasting based on smart meter data, where edge computing supports both privacy and efficiency in distributed environments [2]. The short-term probabilistic load forecasting has also been explored using federated learning, demonstrating effective prediction accuracy and privacy preservation when individual-level data remains decentralized [8]. Privacy-preserving frameworks using edge computing have additionally been applied in Internet of Things (IoT) contexts, enabling secure analytics on sensitive data without transmitting raw information to central servers [3]. In the smart grids, the substantial volumes of consumption data are collected through metering infrastructure, so privacy remains a core concern. Various frameworks have been introduced to protect data confidentiality while ensuring functionality in distributed energy monitoring systems [4].

The federated learning has been employed for electricity theft detection, where secure and decentralized training mechanisms support model development without requiring access to private consumption patterns [5]. Recent advancements also include heterogeneous federated learning frameworks capable of addressing class imbalance, thereby improving detection accuracy in real-world smart grid scenarios while maintaining the privacy of client-side data [14].

To overcome the limitations of centralized learning systems, peer-to-peer federated learning architectures have been adopted, allowing distributed devices to collaborate without a single aggregation server. The secure decentralized training protocols have been demonstrated to be effective in IoT-based settings [6], and alternative designs using peer-to-peer model exchanges are found to be effective in achieving both resilience and scalability [11]. Security enhancements in federated learning have increasingly incorporated homomorphic encryption to enable encrypted model updates to be processed without decryption. The federated learning frameworks with support for homomorphic encryption (HE) have enabled secure model aggregation and privacy-preserving training in edge-based environments [13]. Additional encryption schemes have been proposed that support scalable and efficient encrypted computations using compact ciphertexts in multi-party learning scenarios [15].

Privacy-preserving data aggregation techniques have also been developed using fog computing architectures that incorporate signcryption to protect data during query and aggregation operations [9]. The secure classification models are designed for smart grid applications to achieve a balance between data utility and privacy, supporting accurate and confidential system-level analytics [10]. In parallel, federated learning has been applied to detect intrusions in software-defined networks, showcasing the flexibility of this learning paradigm for broader infrastructure protection [7].

Generative models have also been used in federated learning systems to support privacy-aware data sharing and model robustness when access to raw data is limited [12]. Foundational research in fully homomorphic encryption [16] and federated model aggregation algorithms [17] underpins many of the secure and communication-efficient FL systems currently under development. Building on these foundations, recent work [14] has introduced heterogeneous federated learning frameworks designed for smart grid applications, addressing challenges like data imbalance and non-IID distributions. While these approaches improve detection accuracy in scenarios such as electricity theft, they often lack evaluation under dynamic grid conditions, including fluctuating loads and intermittent connectivity. Validation of federated learning frameworks for smart grids often relies on real-world datasets. Publicly available smart meter datasets from London households have been widely used to evaluate model performance in decentralized energy forecasting scenarios [18,19]. These datasets offer high-frequency and multi-year readings, enabling the design and testing of scalable federated learning solutions suitable for smart grid environments.

### 2.1. Summary of Related Work

To contextualize our contribution, we present a comparative summary of existing research efforts that integrate various technologies within SG and FL frameworks. Table 2 highlights the scope and limitations of recent studies that utilize combinations of FL, P2P networking, EC, HE, and IoT technologies. While many of these studies demonstrate significant progress in privacy-preserving model training, decentralized learning, and IoT-based energy forecasting, none offer a unified framework that simultaneously leverages all five core technologies. This underscores the novelty of our proposed SG IoT framework, which addresses these limitations by fully integrating P2PFL, EC, and CKKS-based HE within an IoT ecosystem for secure and scalable energy consumption prediction.

### 2.2. Research Gaps

While the existing literature (as summarized in Table 2) addresses privacy preservation within machine learning model design, it largely overlooks security considerations across the broader FL pipeline, particularly the protection of inter-client communications and the safeguarding of localized data on individual client devices. Although [14] utilizes CKKS homomorphic encryption to enhance privacy, its reliance on a centralized FL architecture introduces vulnerabilities, such as susceptibility to DDoS attacks and single points of failure. Meanwhile, ref. [15] proposes P2PFL-E, which applies FHE to peer-based aggregation, but restricts model aggregation to a single selected peer at any one time, potentially limiting scalability and resilience. A clear research gap exists in designing an IoT-based smart grid framework that fully integrates P2P federated learning with robust homomorphic encryption to ensure privacy-preserving, decentralized model training. To address this, the present work proposes a novel framework that combines P2PFL and HE techniques for secure and efficient energy consumption prediction in smart grid IoT environments.

## 3. Description of the Proposed Framework

Given the knowledge of the types of FL architecture that can be implemented, we aim to propose an SG IoT framework by combining hierarchical and decentralized FL [1]. The key idea is to use edge devices to perform both model computations for the clients and to communicate with other edge devices in a decentralized manner. All the devices are using end-to-end encryption to ensure that the chain of privacy starts with the client [1].

**Figure 1:**
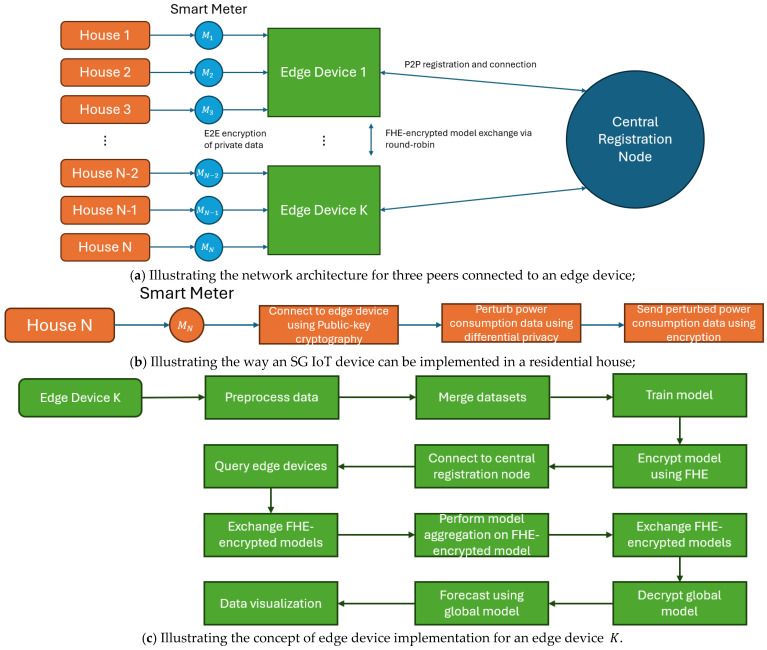
The proposed P2P FL architecture utilizes HE principles.

Our proposed framework is based on P2PFL principles (see Figure 1) in which edge devices connect to each other using P2P networking. The exchanged data between peers is encrypted using CKKS fully homomorphic encryption (FHE). Initially, a peer establishes a connection with a Central Registration Node (CRN) acting as a repository for any peers in the network who can obtain connection details for other peers to be connected to the network. Likewise, any SG IoT device can be connected to specific edge devices using E2E encryption. These devices can act as “sensors” to collect data to be encrypted and transmitted to the edge devices for processing and model data training [11].

### 3.1. Framework Architecture

**Figure 2:**
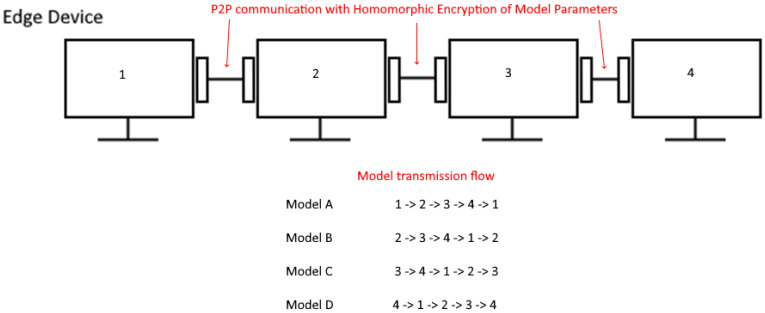
Illustrating the communication pathways between each peer in the P2P network. Each peer exchanges its own localized model with other peers in the network in a round-robin fashion.

**Figure 3:**
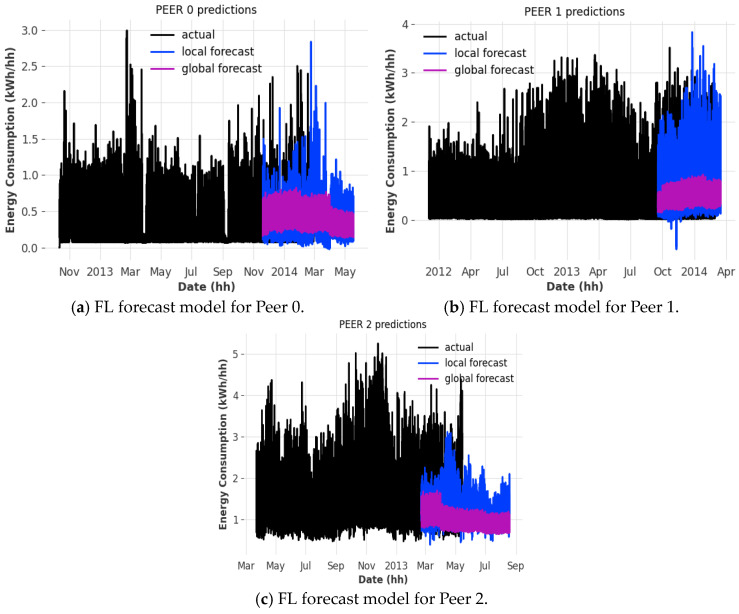
FL forecast model generated using an RNN LSTM using the HE-encrypted model aggregation process.

The proposed framework (Figure 1a) is designed such that each SG IoT device (e.g., Smart Meter) is connected in parallel with other SG IoT devices to an edge device in a secure manner. Each IoT device establishes a connection using public-key cryptography for end-to-end encryption with its respective edge device to send encrypted power consumption data for model training (see Figure 1b). The power consumption data received by the edge device has been perturbed using Differential Privacy (DP) by the IoT device prior to the exchange. For public-key cryptography, we utilize the Hypertext Transfer Protocol Secure (HTTPS), a Transport Layer Security (TLS) protocol; a secure version of Hypertext Transfer Protocol (HTTP) strengthens security by preventing vulnerabilities such as Man-In-The-Middle (MITM) attacks ensuring E2E encryption on both the client and server side (client-to-client in a P2P context). In Figure 1c, each edge device preprocesses the received data and merges the preprocessed data onto a single dataset to feed into an ML model. This model is then trained and encrypted for distribution to other edge devices in the network to construct the global model in a privacy-preserving manner using a modified FL aggregation algorithm with FHE support. The edge device then connects (using HTTPS) to the CRN to both register itself and query other edge devices to exchange FHE-encrypted models in a round-robin fashion (see Figure 2). The model aggregation is then performed on all the exchanged models to find their way back to their originating device, where they are then decrypted, containing the global model in each. This global model is then used to create a forecast of future power consumption in the grid on a per-household Basis, through data visualization (see Figure 3).

### 3.2. Framework Threat Model

The edge device and CRN are honest-but-curious entities as they follow the protocol specifications but may attempt to discern individual households’ power consumption data. The households are fully trusted entities as their smart meters cannot be altered, and data tampered with. The external entities are malicious. External entities may attempt to intercept, read, and modify data to influence the federated power consumption model. In our framework, since DP is applied to perturb the power consumption data from the households, there is no risk of data leakage in any communication between the households and edge devices. Likewise, since the models are homomorphically encrypted during the exchange between edge devices, there is no risk of data leakage in any communication between the edge devices and CRN.

## 4. Theoretical Contribution to Fully Homomorphic Encryption

The theoretical foundation of our work is the development of a smart grid IoT framework by integrating P2PFL architecture and homomorphic encryption (HE) principles. The proposed framework is discussed in detail in Section 3. Another theoretical aspect of our work is the development of a fully HE technique. To this end, we mathematically derive efficient FHE encryption (see Equations (1)–(6)).

Specifically, we utilize the CKKS encryption scheme [12] to encrypt floating-point values (used heavily by tensors, which represent the model parameters as matrices). The reason we opt to use the CKKS scheme over the Brakerski–Gentry–Vaikuntanathan (BGV) encryption scheme is. The CKKS scheme is much better at efficiently encrypting floating-point values, which are allowed to accumulate minor errors, resulting in approximate value encryption [16]. While BGV is better suited for cases where numerical accuracy must be preserved throughout the encryption stage, which severely limits plaintext storage under hard memory constraints. Because of these differences, CKKS is better suited for encrypting values such as model parameters, as numerical accuracy is not of utmost importance in the case of ML workloads. The BGV is better suited to ensuring plaintext integrity. Equation (1) transforms a tensor from a matrix form to a vector form using the get_tensor_as_vector function for more efficient FHE encryption.

(1) T=T00…T0(n−1)⋮⋱⋮Tm−10…T(m−1)(n−1)→get_tensor_as_vectorT⃑=(T00…Tm−1n−1)ST⃑=(m,n)

To perform FHE encryption on an entire ML model, we need to consider encrypting all relevant model parameters to ensure that no malicious actors can intercept and decipher any weight values, which could lead to data leakage in a secure setting. To achieve this, we perform encryption on all tensors that represent the model parameters in an ML model. However, this can be a very expensive operation to perform on tensors with dimensions larger than 784 × 69. Initial test results show that the memory usage skyrockets when attempting to encrypt tensors of these dimensions, which consume an excess of 4 GB, and beyond which would not be suitable for workloads carried out with especially large models with numerous parameters. For optimization, we derive a mathematical model (see Equation (1)) to transform each tensor using a transformation function which maps a tensor T to a vector $\overset{⃑}{T}$ and stores the shape in a separate vector $\overset{⃑}{S_{T}}$. This allows us to invert this mapping transformation later when we decrypt the encrypted tensor by first creating a 1-dimensional tensor which is then re-shaped using the encoded shape information that was derived from the initial encryption step.

It is vital that the shape information is kept and exchanged, as it is the only way that a tensor can be restored via inverse mapping once the initial mapping transformation has occurred. In this case, each peer has the same model hyperparameter, which specifies the number of parameters present in the model as well as the shape of each tensor that represents these parameters. We do not need to worry about encrypting the shape information as there is no concern of data leakage of any of the parameters, which would contain sensitive values during model aggregation. Equation (2) performs vector addition on an encrypted tensor $\overset{⃑}{T}$ with an unencrypted tensor U, which has been transformed to a vector $\overset{⃑}{U}$ temporarily using the mapping function defined in Equation (1).

(2) $$\overset{⃑}{T}=\overset{⃑}{T}+\overset{⃑}{U}$$

Each peer generates a public key $P_{k}$ and a secret key $S_{k}$ and encrypts model parameters P with its own $P_{k}$. $E\left(p\right)=C$, the ciphertext C is then exchanged with other peers (in turn retrieving their ciphertexts containing their model parameters) and can perform a variation in FHE to sum and multiply (divide using inverse multiplication) to each compute the global model parameters $C^{\prime }$. Equation (3) computes a shared ciphertext $C^{\prime }$ given a list of ciphertexts with a specific order of transformations (i.e., addition). In Equation (4), we compute the shared model ‘S’ by decrypting the shared ciphertext $C^{\prime }$ and finding the average of n models, where n is the number of peers. These transformations in this example represent computing the global model ciphertext using the Federated Averaging (FedAvg) algorithm [17].

(3) $$C^{\prime }=\sum_{i=0}^{n}C_{i}$$

(4) $$S=\frac{D(C^{\prime })}{n}$$

Once all the tensors representing the model parameters have been encrypted, we can then transmit these now encrypted tensors in a round-robin fashion to each peer within the P2P network and perform aggregation via the FedAvg algorithm with a different approach [17]. We first perform addition of each peer’s local model parameters with the encrypted tensors (using Equation (3)) representing a certain peer’s local model parameters, with each peer passing the encrypted model forward to another peer in round-robin fashion (see Figure 2) until the encrypted model returns to the initial peer that made an aggregation request in the network. The initial peer can then decrypt the encrypted model, $D\left(C^{\prime }\right)=P^{\prime }$ where $P^{\prime }$ represents the sum of all peer model parameters in a decrypted state, which will now contain tensors which represent the sum of all model parameters in the P2P network. We then divide each of these tensors by the number of additional operations performed (see Equation (4)) on the encrypted tensors to determine the global FL model without the risk of model data leakage occurring at any stage in the aggregation process within the network while the model is in possession by a different peer. This process is repeated on each peer within the network, allowing each peer to derive the same global model while maintaining both security and privacy within the network.

## 5. Performance Evaluation

In this section, we discuss the system performance evaluation, including simulation environment, hyperparameter optimization, and model validation. The simulation environment is discussed next.

### 5.1. Simulation Environment

To evaluate the effectiveness of our FL framework, we use a Python-based implementation in conjunction with the dataset described in Section 5. This provides a suitable simulation environment replicating a real-world scenario in which the framework would be used. Training is performed on an NVIDIA Graphics Processing Unit (GPU) utilizing Tensor Cores to improve training performance during the simulation due to the amount of time it can take to train the same model in a Central Processing Unit (CPU), using the PyTorch 2.7.0 CUDA package. Additionally, the tuned model parameters are provided (see Table 3).

**Figure 4:**
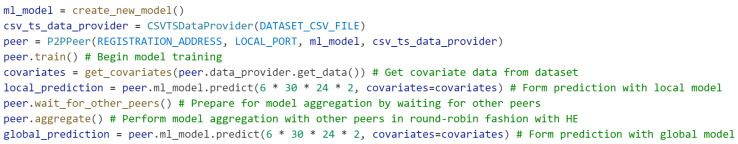
Sample pseudocode to train an FL model for any number of peers using P2P.

To evaluate the FL framework, a simulated network is established by running multiple clients with different datasets on a local machine in a P2P configuration abstracted using the CRN as a central repository to distribute connectivity details between each client. The SG dataset used for evaluating the proposed IoT framework in this paper is based on household electrical power consumption measured by smart meters in London [18]. The dataset comes with smart meter reading data captured from 5567 households in London between November 2011 and February 2014, in half-hourly, hourly, and daily sets of data, which allows for a flexible approach when testing the FL framework with an ML model [19]. In this case, the half-hourly dataset was used to train and predict daily trends in power consumption for households that are connected to the simulated SG system utilizing the FL framework, due to a greater amount of data present compared to the hourly and daily sets. A preprocessing step is run on the data to eliminate any null values, which can cause undefined behavior during model training. A combination of forward and backward filling is used to interpolate and fill in gaps between data points (typically a missing half hour). The data is scaled using a min-max scaler mapped to the ranges $\left[0\leq n\leq 1\right]$ fitted to the training set, to ensure even distribution of data during the model training process. This mapping is reversed during the forecast prediction phase using inverse mapping. The ML model used is an RNN model using the Long Short-Term Model (LSTM) sub-model. We generate covariate data (year, month, day, minute, and quarter) to supply alongside the initial dataset with time series beginning from the first timestamp to the last, with a couple of extra data points for the six-month prediction window. The year, month, day, hours, and minute covariate series use one-hot encoding, while the quarter covariate series uses cyclical encoding to allow the model to better map data points to different parts of the year consistently. Finally, we stack all the individual covariate series, which we then supply to the RNN model as future covariates. We also opted to use the FedAvg algorithm for FL model aggregation [17]. The FedAvg algorithm is a common algorithm that is used in FL when aggregating with various trained client models. These models can be replicated with a version that supports HE, as pointed out in [13], thanks to the homomorphism property of HE [17]. Figure 4 shows a sample algorithm pseudocode using P2P to train an FL model for any number of peers.

An example of algorithmic code utilizing P2P is shown in Figure 4. To demonstrate using the framework to train an FL model among numerous peers sharing a CRN using a P2P architecture. In this example, the CSV file is provided as the first parameter through the command line when executing the example program. The P2PPeer ‘start’ function is then called, which registers the peer to the provided registration server at REGISTRATION_ADDRESS with the port LOCAL_PORT for other peers to connect to via the registration server. The peer then performs local model training using the dataset provided, generating a prediction using the locally trained model, and then waits for other peers to do the same via the registration server using the wait_for_other_peers function. Once the wait_for_other_peers function returns, indicating that all other connected peers have performed local model training, all connected peers then individually perform global model aggregation and distribution via the aggregate function. Once the global model has been finalized, the program generates another prediction using the global model.

### 5.2. Hyperparameter Optimization

Hyperparameter optimization is performed using the Ray Tune Python 3.13.3 library to test various specified model hyperparameters. This optimization allows us to find a good combination of values to tune the model and produce more accurate predictions with a lower validation loss during training. It allows us to train multiple models with varying hyperparameters in parallel with the same subset of data provided to each trial. In other words, models of the same kind with varying hyperparameters are fine-tuned models using the FL framework to generate SG predictions with both local and global forecasts for each peer. Once all trials run and results are obtained (see Table 4), one can feed the best hyperparameters found with the least validation losses into our generalized model.

### 5.3. Model Validation

**Figure 5:**
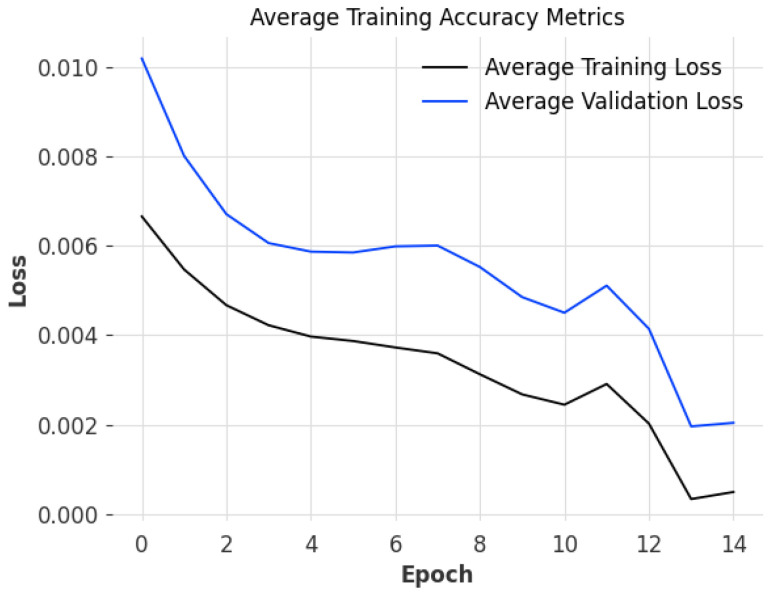
The training accuracy metrics averaged over 100 peers produced as part of training multiple localized RNN LSTMs on half-hourly data to aggregate into a global model.

The system model is validated by comparing training accuracy metrics (Figure 5) to ensure that the trained model parameters were optimal given the datasets provided. When training a model, great care needs to be taken when tuning model hyperparameters, as it is easy to underfit or overfit a model, which results in noisy and inaccurate predictions. Underfitting a model causes bad predictions as the model is unable to establish a relationship between inputs and outputs from the training dataset. Likewise, overfitting a model causes the opposite effect, where a model will attempt to apply familiar relationships in a repetitive manner by looking at patterns in the data instead of learning the nature of these relationships from the data. To prevent underfitting and overfitting from occurring, we use early stopping, which determines the most recent epoch where the difference between the training loss and validation loss was the most minimal, which is deemed to be the best fit for the model. By ensuring that we use the best fit for the models, we can have high confidence that the predictions that are produced by the models and the global model produced following aggregation are as accurate as possible. This is especially true given that we utilize model hyperparameter optimization to ensure that the initial hyperparameters reduce the initial losses at the beginning of the process.

## 6. Results and Discussion

**Figure 6:**
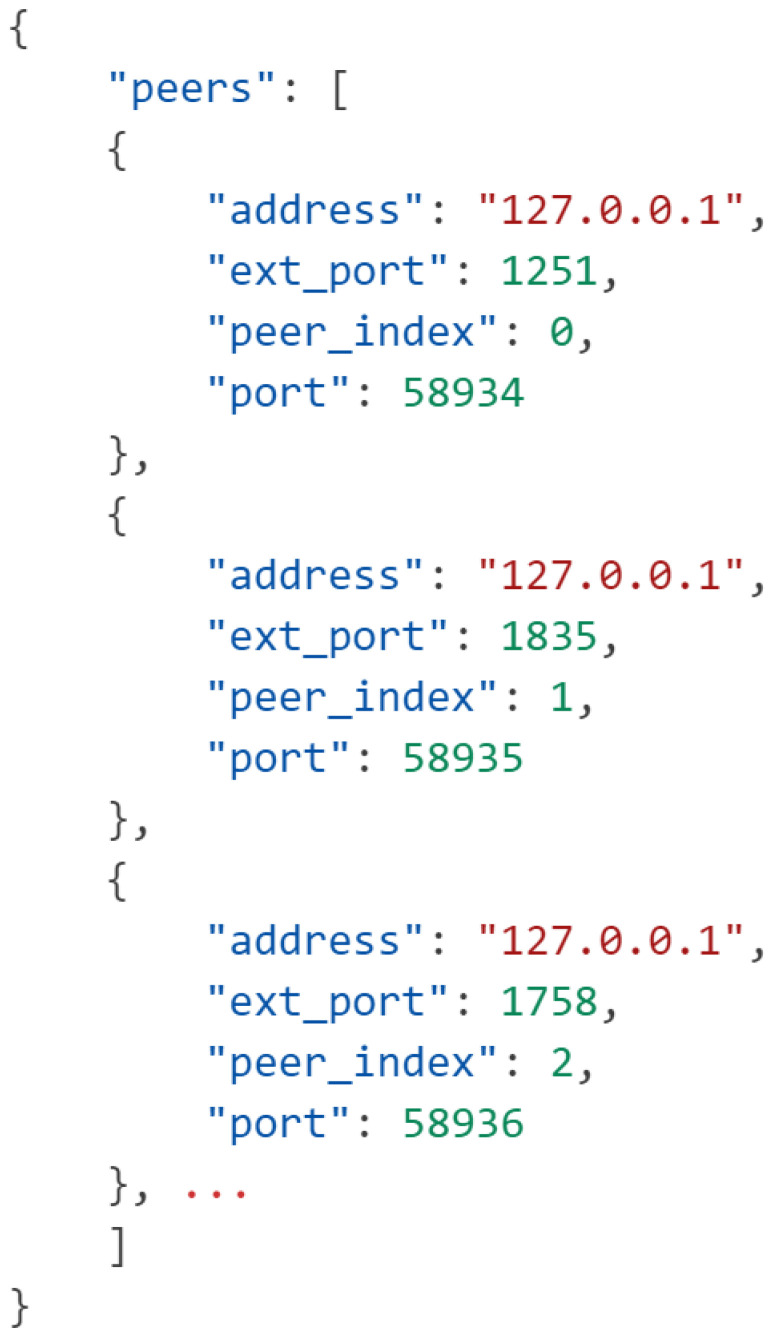
JSON data is sent to each P2P client from the CRN, providing clients enough information to connect to each other without revealing any identifying information.

We observe that the framework can successfully compute an FL model from 100 separate ML models running on separate instances communicating through the previously established P2P architecture (see Figure 6). The CRN registers each peer’s address and port from which a connection was established and receives the communication port from the client itself. Additionally, the CRN determines each peer’s index relative to each other to streamline the model aggregation process later. Because the P2P architecture does not require much information to establish, we eliminate any personally identifiable information that can be used to connect a client to a data source, thus preserving user privacy. The average runtime per peer is 25 s, with 0.08% of that time spent on performing CKKS FHE, and 82.66% of that time being spent on training local models, leaving 17.26% of that time being spent on model aggregation and prediction.

On scalability, we validate the system’s performance for 100 peers. The proposed framework can be scaled up to thousands of peers sharing one CRN; noting, however, that network congestion between peers would become a huge issue due to our round-robin approach of model aggregation. In simulation, the model parameters occupy around $S_{M}≅2$ MB after encryption and encoding. For distributed across n=100 peers during model aggregation with the round-robin approach.

For $S_{T(P)}≅200$ MB of model data that will be transmitted per peer (Equation (5)). Extrapolating this number to all 100 peers, we would look at network transmission costs of up to $S_{T}=20$ GB within the network (Equation (6)).

(5) $$S_{T\left(P\right)}=S_{M}*n$$

(6) $$S_{T}=S_{T(P)}*n=S_{M}*n^{2}$$

One can observe that the FL model can predict power consumption trends to some accuracy, depending on the client that it is running, compared to the local forecast model (similar trends and patterns depending on the date) (see Figure 3).

We can observe that the individual losses of the localized models we train before aggregation occur (see Figure 5) hover between 0.1% to 1%, given the simulation parameters provided when training and validating the model against the test dataset. As with all cases with FL models, the number of models that participate in the FL model aggregation process, the more accurate the FL model. Another thing to note is that each peer has been trained against the same hyperparameters (see Table 3), which can result in non-optimal model performance due to varying data patterns in different datasets, which can cause dramatic fluctuations in the output of the FL model [6]. A potential solution to this issue would be to use adaptive hyperparameter optimization, where each peer derives a set of hyperparameters from past data, which can then be used to train a model with better accuracy [6]. Furthermore, this will enhance the performance of the global model as individual hyperparameters only affect model parameters during the training process.

### 6.1. Practical System Implications

**Figure 7:**
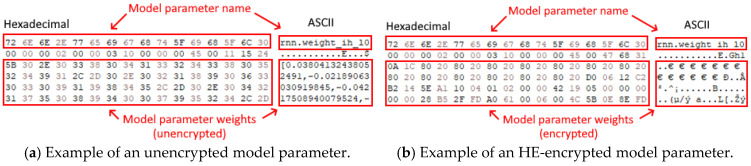
A comparison of the unencrypted local client model (**a**) and the HE-encrypted client model (**b**) that is distributed to other P2P clients for FL model aggregation.

To ensure that the implementation of HE functions as expected, we can observe that a comparison between the model data stored locally on a client and the same model data fetched from another client shows differing payloads by comparing the binary output in hexadecimal form. By looking at Figure 7, one can observe that the model parameter “rnn.weight_ih_10” is encrypted from its original value using HE, which is then distributed to other clients in the P2P network in which each client will then average their own weights against the received encrypted models from other clients using FedAvg to derive the FL model securely, preventing any data leakage from occurring from model data.

Network planners can benefit from the P2P architecture that the framework utilizes by reducing communication delays between peers through direct connection instead of through a middleman centralized server, as well as reducing cost by requiring less infrastructure. This does, however, come with a catch, as individual peers will need to be equipped with enough computational power to perform ML workloads in a reasonable amount of time. However, the cost will ultimately depend on whether a fast and performant output is required, sacrificing power efficiency and cheap costs, and vice versa. In both cases, however, privacy preservation remains a key benefit of utilizing a P2P architecture in this case, as it becomes harder to attack a decentralized network instead of one relying on a central authoritative host. In case of a DDoS attack, the single point of failure in a P2PFL network is the CRN. Should the primary CRN be taken offline, backup CRNs can be on standby, ready to take over as each peer has a list of addresses of backup registration nodes in numbered order to poll and connect to.

**Figure 8:**
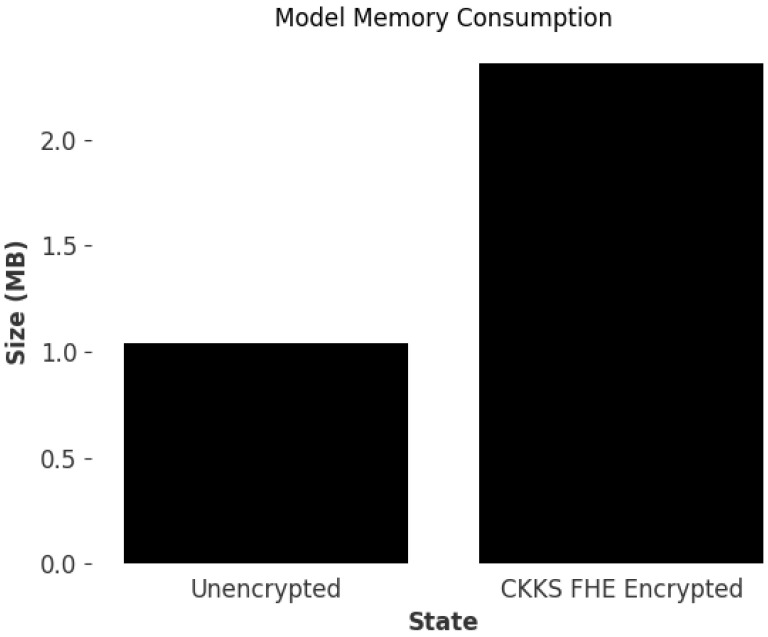
Memory consumption. Comparison between unencrypted encoded model and the CKKS FHE-encrypted encoded model.

Following HE, the memory footprint of the encrypted model parameters increases by 126% (see Figure 8) due to the additional data needed to support decryption and evaluation of the ciphertext. In the context of IoT devices, this additional footprint could be reduced by breaking up model parameters and sending chunks of parameters for aggregation between peers, allowing reconstruction of parameters once the round-robin cycle is completed, reducing memory consumption from HE.

### 6.2. Open Issues and Future Directions

While our proposed framework uses CKKS for single-key encryption, extending the framework to support MKFHE would enable secure aggregation across decentralized peers without requiring a shared secret key. This would eliminate the round-robin ciphertext exchange bottleneck and reduce network bandwidth saturation in large-scale P2P networks. Future work will explore MKFHE schemes combined with adaptive quantization techniques to minimize ciphertext size, enabling efficient multi-party computations on resource-constrained edge devices.

To address latency introduced by HTTPS/TLS in large networks, we plan to integrate lightweight cryptographic primitives (e.g., lattice-based signatures) with hardware-assisted trusted execution environments (TEEs) for peer authentication. This hybrid approach would balance security robustness with computational efficiency, particularly for time-sensitive smart grid applications. The memory overhead of CKKS remains a barrier for low-end IoT devices. Future studies will optimize HE operations for edge hardware by leveraging model pruning, federated dropout, and FPGA-accelerated encryption kernels. Collaborations with SG testbeds (e.g., IEEE PES GridLAB-D) will validate these optimizations in the real-world grid conditions, including dynamic load fluctuations and intermittent connectivity. Integrating the framework with SG testbed and adopting multi-key FHE (MKFHE) for scalable aggregation is also suggested as future work.

Moreover, in practical applications, SG systems often encounter dynamic load fluctuations and intermittent connectivity, which can disrupt P2P communication and affect FL model performance. To address these issues, future iterations of the framework will explore adaptive load-aware scheduling and asynchronous FL strategies to reduce sensitivity to fluctuating data availability and network instability. Integrating buffering and retry logic at the edge level can further enhance robustness against temporary disconnections.

## 7. Conclusions

In this paper, we proposed a robust smart grid IoT framework by integrating FL, EC, and HE, for proving a real-world application of P2PFL for forecasting smart grid data to predict energy consumption patterns. Full encryption of the model parameters of the FL model with HE was achieved to allow privacy preservation of sensitive data and prevent data leakage in a peer-to-peer networking environment while maintaining model accuracy during the end-to-end encryption process. Results obtained have shown that the accuracies of the localized models were up to 98% when training and validating the model against the test dataset. Future research work includes developing a new derivative HE scheme where a shared private key can be generated from a set of public keys plus a given peer’s secret key to decrypt a shared ciphertext, given that similar transformations are applied to both the ciphertext and the key. We uncovered a novel encryption scheme, Multi-Key Fully Homomorphic Encryption (MKFHE), which allows multiple ciphertexts to be grouped into one generalized ciphertext to allow transformations to be performed on all grouped ciphertexts at once [15]. However, MKFHE does not address the need for each peer to be able to independently perform these transformations locally, as it still requires each peer to exchange encrypted models in a round-robin fashion, which risks saturating network bandwidth in cases where many peers may need to perform model aggregation to compute the global model [15].

## Figures and Tables

**Table 1:** List of abbreviations used in this paper.

Abbreviation	Notation
BGV	Brakerski-Gentry-Vaikuntanathan
CKKS	Cheon–Kim–Kim–Song
CPU	Central Processing Unit
CRN	Central Registration Node
CSV	Comma-Separated Values
DDoS	Distributed Denial of Service
DP	Differential Privacy
E2E	End-to-End
EC	Edge Computing
FHE	Fully Homomorphic Encryption
FL	Federated Learning
GPU	Graphics Processing Unit
HE	Homomorphic Encryption
HTTP	Hypertext Transfer Protocol
HTTPS	Hypertext Transfer Protocol Secure
IoT	Internet of Things
JSON	JavaScript Object Notation
LSTM	Long Short-Term Memory
MITM	Man-In-The-Middle
MKFHE	Multi-Key Fully Homomorphic Encryption
ML	Machine Learning
MSE	Mean Squared Error
P2P	Peer-to-Peer
P2PFL	Peer-to-Peer Federated Learning
RNN	Recurrent Neural Network
SG	Smart Grid
TLS	Transport Layer Security

**Table 2:** Summary of related work on SG FL IoT framework.

Reference	Technologies Used						Key Contributions
	FL	P2P	EC	HE	IoT	Blockchain	
Abdulla, et al. [2]	✓	✕	✓	✕	✓	✕	Introduces an adaptive FL model using EC architecture which improves privacy preservation, reduces error rates, and speeds up training times in SG IoT.
Nair et al. [3]	✓	✕	✕	✕	✓	✕	Proposes a privacy-preserving FL framework for IoMT using edge computing, ensuring compliance with GDPR.
Hijazi et al. [6]	✓	✓	✕	✕	✓	✕	Introduces secure P2P FL for IoT collaboration, enabling decentralized model aggregation without a central server.
Liu, et al. [8]	✓	✓	✓	✕	✕	✕	Improves model training by distributing SG forecasting models instead of distributing user data to improve privacy preservation.
Li et al. [9]	✕	✓	✓	✓	✕	✕	Designs a fog computing-based privacy-preserving scheme for smart grids, enabling secure data querying and aggregation.
Singh et al. [10]	✓	✓	✕	✕	✓	✕	Develops a secure data aggregation and classification model for smart grids, balancing privacy and utility.
Roy et al. [11]	✓	✓	✓	✕	✕	✕	Introduces Brain Torrent, a decentralized P2P FL architecture for medical data sharing without centralized coordination.
Chen et al. [12]	✓	✓	✓	✕	✓	✕	Proposes Federated-WDCGAN, a federated smart meter data sharing framework that enhances privacy preservation without sacrificing data utility.
Zeng et al. [13]	✓	✕	✕	✓	✓	✕	Proposes a federated learning framework with CSP-based HE for secure model aggregation in edge computing.
Wen et al. [14]	✓	✕	✕	✓	✕	✕	Introduces a novel data partitioning and aggregation scheme that detects SG energy theft patterns on encrypted data, leveraging CKKS FHE.
Zhou et al. [15]	✓	✕	✕	✓	✕	✕	Proposes a compact multi-key FHE scheme to enable scalable encrypted data analytics.
Brakerski et al. [16]	✓	✕	✕	✓	✕	✕	Introduces a foundational approach to FHE enabling operations on encrypted data without bootstrapping, establishing groundwork for secure ML frameworks.
McMahan et al. [17]	✓	✕	✕	✕	✕	✕	Introduces a communication-efficient FL algorithm (FedAvg) that reduces communication overhead by averaging model updates across clients.
Mao et al. [20]	✓	✕	✓	✓	✓	✓	Proposes a Blockchain-based approach to validating smart meter data for performing FL workloads in SG using EC.
Our work	✓	✓	✓	✓	✓	✕	Proposes P2PFL EC architecture when forecasting energy consumption in SG IoT using secure peer-peer model aggregation on CKKS FHE-encrypted model data.

**Table 3:** Parameters used in the simulation.

Parameter	Value
No. of peers	100
ML model	RNN
FL algorithm	FedAvg
RNN model	LSTM
Hidden neuron count	64
RNN layer count	1
RNN dropout	0
Batch size	1024
Input size	48
Sequence length	49
Epochs	15
Optimizer	Adam
Parameter Count	50.5 K
Learning rate	0.01
Loss function	Mean Squared Error (MSE)
Early stopping monitor	Validation Loss
Early stopping patience	5
Early stopping min delta	0.0005
Early stopping mode	min
Training set to validation set ratio	4:1

**Table 4:** Hyperparameter optimization using Ray Tune to determine which combination produces the least validation loss (the “loss” column) on a given subset of data. The first 10 results are shown with values rounded to two decimal places.

Batch_Size	N_Rnn_Layers	Dropout	Input_Chunk_Length	Hidden_Dim	Loss_Fn	Iter	Total Time (s)	Loss
64	3	0.05	32	114	MSELoss()	6	29.82	0.02
64	1	0.09	64	210	L1Loss()	3	23.03	0.08
64	1	0.23	64	123	MSELoss()	6	31.39	0.02
64	3	0.34	32	224	L1Loss()	3	35.77	0.09
128	3	0.10	32	95	L1Loss()	3	10.52	0.08
16	2	0.43	16	242	L1Loss()	3	34.23	0.08
128	3	0.08	32	68	MSELoss()	6	20.58	0.02
64	3	0.26	128	96	MSELoss()	6	62.65	0.02
128	2	0.18	128	242	MSELoss()	6	164.95	0.02
256	1	0.38	128	145	L1Loss()	3	21.15	0.07
…	…	…	…	…	…	…	…	…

## Data Availability

The original contributions presented in this study are included in the article. Further inquiries can be directed to the corresponding author.
